# Oxidative Stress-Mediated Atherosclerosis: Mechanisms and Therapies

**DOI:** 10.3389/fphys.2017.00600

**Published:** 2017-08-23

**Authors:** Xinyu Yang, Yang Li, Yanda Li, Xiaomeng Ren, Xiaoyu Zhang, Dan Hu, Yonghong Gao, Yanwei Xing, Hongcai Shang

**Affiliations:** ^1^Guang'anmen Hospital, Chinese Academy of Chinese Medical Sciences Beijing, China; ^2^Key Laboratory of Chinese Internal Medicine of the Ministry of Education, Dongzhimen Hospital, Beijing University of Chinese Medicine Beijing, China; ^3^Department of Cardiology, General Hospital of People's Liberation Army Beijing, China; ^4^Masonic Medical Research Laboratory Utica, NY, United States

**Keywords:** oxidative stress, atherosclerosis, inflammation, apoptosis, mitochondria, autophagy, epigenetics, therapies

## Abstract

Atherogenesis, the formation of atherosclerotic plaques, is a complex process that involves several mechanisms, including endothelial dysfunction, neovascularization, vascular proliferation, apoptosis, matrix degradation, inflammation, and thrombosis. The pathogenesis and progression of atherosclerosis are explained differently by different scholars. One of the most common theories is the destruction of well-balanced homeostatic mechanisms, which incurs the oxidative stress. And oxidative stress is widely regarded as the redox status realized when an imbalance exists between antioxidant capability and activity species including reactive oxygen (ROS), nitrogen (RNS) and halogen species, non-radical as well as free radical species. This occurrence results in cell injury due to direct oxidation of cellular protein, lipid, and DNA or via cell death signaling pathways responsible for accelerating atherogenesis. This paper discusses inflammation, mitochondria, autophagy, apoptosis, and epigenetics as they induce oxidative stress in atherosclerosis, as well as various treatments for antioxidative stress that may prevent atherosclerosis.

## Introduction

Atherosclerosis, the formation of atherosclerotic plaques, remains a major reason of the morbidity and mortality in both developed and developing nations (Townsend et al., 2015). The World Health Organization redounds an estimated 16.7 million deaths to the atherosclerotic cardiovascular disease (Association, 2007; Leopold and Loscalzo, 2008). Atherosclerotic plaque rupture is a usual reason of the cardiovascular diseases, as stroke and myocardial infarction (Grootaert et al., 2015). Atherogenesis is a complicated course that concerns some mechanisms including endothelial dysfunction, neovascularization, vascular proliferation, apoptosis, matrix degradation, oxidative stress, inflammation, and thrombosis (Hansson, 2005). The pathophysiological mechanisms of atherosclerosis have yet to be illuminated, though most hypotheses about its pathogenesis and progression concern the disruption of normal homeostatic mechanisms incurring oxidative stress.

Studies have shown that oxidative stress is a pivotal feature of the atherogenesis (Witztum and Berliner, 1998). It is widely defined as the redox status realized when an imbalance exists between antioxidant capability and activity species including reactive oxygen (ROS), nitrogen (RNS) and halogen species, non-radical as well as free radical species (Leopold and Loscalzo, 2009). These conditions cause cell injury by directly oxidizing cellular protein, lipid, and DNA or via cell death signaling pathways (Leopold and Loscalzo, 2009; Sinha et al., 2013). In the cell, ambient levels of certain ROS are used as signaling molecules to sustain fundamental cellulate functions. In comparison, reactivity oxidants and free radicals are produced in absence of the physiological stimulus, and then small molecule antioxidants are depleted or antioxidase systems are being overwhelmed (Leopold and Loscalzo, 2009). It triggers a net increase in the oxidative stress and biologically activated ROS. It not only plays a important part in pathology of the cardiovascular diseases, but also has physiological functions that may adjust cardiomyocytes (Santos et al., 2011). Atherosclerosis is considered as a complex process featured by the positive involvement of immune systems (Galkina and Ley, 2007; Weber et al., 2008; Libby et al., 2013). This study focuses on the method by which inflammation, mitochondria, autophagy, apoptosis, and epigenetics induce oxidative stress to accelerate atherosclerotic lesion formation. Several drug-based treatments for antioxidative stress are also discussed below.

## Reactive oxygen species (ROS)–producing systems in atherosclerosis

ROS at medium concentrations play important signaling roles under various physiological conditions (Li et al., 2014; Förstermann et al., 2017). Excessive ROS production outpacing the usable antioxidant systems results in oxidant stress (Li et al., 2014). Several primary ROS-producing systems are present in blood vessel wall embracing xanthine oxidase (XO), uncoupled endothelial nitric oxide synthase (eNOS), enzymes of the mitochondrial respiratory chain, and nicotinamide adenine dinucleotide phosphate (NADPH) oxidase (NOXs) (Brandes and Kreuzer, 2004; Förstermann, 2008, 2010; Li et al., 2014; Xia et al., 2017). These oxidases, composing of two membrane-combined subunits and several cytoplasmic modulatory subunits, are multisubunit enzyme compoundes which produce superoxide from the molecular oxygen employing NADPH served as electron donor (Bedard and Krause, 2007; Cave, 2009; Drummond et al., 2011). Be contrary to Nox1 and Nox2, Nox4 only needs p22phox and liberates hydrogen peroxide rather than superoxide (Schröder et al., 2012). Three Nox isotypes are expressed in the blood vessel wall of mice with in the vascular smooth muscle cells (VSMC); and Nox2 (Görlach et al., 2000) and Nox4 (Ago et al., 2004; Xu et al., 2008) are primarily expressed in endotheliocytes.

**Figure 1:**
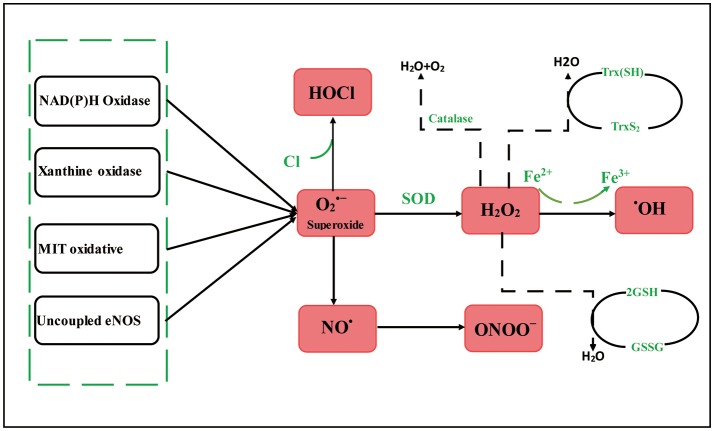
Reactive oxygen species–producing systems in atherosclerosis. MIT oxidative, Mitochondrial oxidative; eNOS, endothelial nitric oxide synthase; ${\text{O}}_{2}^{\;•-}$, superoxide; OX, xanthine oxidase; NO^•^, nitric oxide; HOCl, hypochlorite; H_2_O_2_, hydrogen peroxide; ONOO^−^, peroxynitrite; ^•^OH, hydroxyl radicals; SOD, enzyme superoxide dismutase; GSH, glutathione; Trx, thioredoxin. ${\text{O}}_{2}^{•-}$ can be generated in the blood vessel wall by NOXs, uncoupled eNOS, OX, and mitochondrial respiration chains. H_2_O_2_ can traverse spontaneous transformation to ^•^OH by Fe reaction, SOD. H_2_O_2_ can be detoxified through GSH peroxidase, Trx peroxidase, and catalase to H_2_O and O_2_. Meanwhile, the myeloperoxidase enzyme can employ H_2_O_2_ to oxygenize chloride to the strong oxidizer HOCl. The uncoupling eNOS decreases endothelial NO production, which is further aggravated by reduced eNOS expression and activity.

Recent a study had indicated that Nox enzymes play different roles in atherogenesis (Fulton and Barman, 2016). XO produces hydrogen peroxide and superoxide by employing molecular oxygen as an electron acceptor (Nishino et al., 2008; Nomura et al., 2014). The expression of endothelial XO are increased through proatherosclerotic stimuli like angiotensin II (Ang II) treatment (Landmesser et al., 2007) as well as oscillatory shear stress (McNally et al., 2003). Usually, mitochondrial oxidative phosphorylation generates physiological levels of superoxide which translates into hydrogen peroxide by the manganese-dependent superoxide dismutase (SOD2), as well as subsequently by the glutathione peroxidase 1 (GPx1) to water (Wang et al., 2014a; Phaniendra et al., 2015). Atherosclerosis in humans has been associated with mitochondrial oxidative stress (Corral-Debrinski et al., 1992). eNOS generates NO under certain physiological conditions and thus represents a crucial vasoprotective element for the endothelium (Li and Förstermann, 2000, 2009; Förstermann and Sessa, 2012; Li et al., 2014). Under pathological conditions linked to oxidative stress, however, eNOS may become dysfunctional (Förstermann, 2008; Li and Förstermann, 2013; Li et al., 2013; Figure 1).

## Macrophages induce oxidative stress in atherosclerosis

Macrophages are diverse, bactericidal, and scavenging tissue-resident cells responsible for an array of crucial immune functions (Maiuri et al., 2013). Macrophages are the most numerous immune cell genre in the pathological changes of atherosclerotic, they are concerned from lesion initiation to plaque rupture, and play a requisite role through all stage of the disease (Cochain and Zernecke, 2017). These immune cells mainly consume poisonous blood fat, such as oxidized low-density lipoprotein (ox-LDL) as portion of their normal scavenging function. The lipid-laden macrophages are deposited underneath the endothelium of arteries, eventually forming obstructive atherosclerotic plaques. Recent studies have confirmed that macrophage cellular oxidation, 7-hydroperoxide (7-OOH), autophagy protein 5 (ATG5), and thiol oxidative stress enhances macrophage cellular oxidative stress and accelerates atherosclerotic plaque progression.

### Macrophage cellular oxidation and oxidative stress

A recent study (Abu-Saleh et al., 2016) demonstrated that the components of atherosclerotic plaque enhance macrophage cellular oxidation. Macrophages from atherosclerotic normo- or hyper-glycemic apoE^−/−^ mice were cultured with mouse aorta aqueous or lipid extract dated from these mice, while J774A.1-incubated macrophages were cultured with enhancing concentrations of extracts prepared from the human carotid atherosclerotic lesion: hydrophobous injury lipid extract, human body injury aqueous extract, or the conjunction of two. Macrophage oxidative status, triglyceride, and cholesterol metabolism were analyzed over the course of the experiment to find that aqueous and lipid extracts markedly enhanced the oxidative stress of macrophages (Lowry et al., 1951; Meir and Leitersdorf, 2004; Abu-Saleh et al., 2016). Compensatory enhances in the cellular antioxidant reagent paraoxonase 2 (PON2) activity and the macrophage glutathione was viewed after cultivation with all extracts (Lowry et al., 1951; Gaidukov and Tawfik, 2005). And macrophage triglyceride biosynthesis rate and mass enhanced dramatically with treatment in the lipid extracts and the upregulation of diacylglycerol acyltransferase (Abu-Saleh et al., 2016). These extracts resulted in a decrease in the cholesterol biosynthesis rate by the downregulation of HMG-CoA reducase and the limiting velocity enzymes in the cholesterol biosynthesis (Thomas et al., 2008). This above findings demonstrated that the interreaction among kinds of lesion extracts and macrophages can help in the atherosclerosis development by enhancing macrophage oxidation and lipid cumulation, bringing about the formation of foam cells.

### 7-hydroperoxide (7-OOH) and oxidative stress

Oxidative stress related to cardiovascular system disease is able to generate all kinds of oxidized lipids embracing cholesterol oxidations as 7-OOH, 7-ketone (7=O), and 7-hydroxide (7-OH) (Brown et al., 1997; Brown and Jessup, 2009). The stimulation of human monocyte-originated THP-1 macrophages with dibutyryl-cAMP basically upregulates StarD1 and ABCA1 (Hakamata et al., 1998). In previous study, SiRNA-induced StarD1 knockdown preceding to stimulation did not influence StarD4 but brought down ABCA1 upregulation, and the latter is related with StarD1 function (Hakamata et al., 1998; Borthwick et al., 2009). Compared with non-stimulated controls, mitochondrion with the stimulated StarD1-kd cells innerized 7-OOH more slowly and went through less 7-OOH-induced membrane depolarization and lipid peroxidation, like determined by C11-BODIPY and JC-1 probes (Ma et al., 2007). The primary functional outcomes of 7-OOH exposed are: (1) reduced 27-hydroxycholesterol (27-OH) output, (2) forfeit of mitochondrial 27-OH by the activity of 27-hydroxylase (CYP27A1), and (3) reduction of the cholesterol-exporting ATP-binding cassette and ABCA1 subfamily G member 1 (Korytowski et al., 2013). Similarly, compared with non-challenged macrophage controls, challenged macrophages export fewer cholesterol to apolipoprotein A-I or HDL (Hakamata et al., 1998; Brown and Jessup, 2009). Previous researchers (Korytowski et al., 2015) identified the mechanism through which macrophage cholesterol efflux can be lost ability under oxidative stress-related disease like atherogenesis. Their findings also revealed the effect of macrophage redox disorders in the atherogenesis.

### ATG5 and oxidative stress

Macrophage apoptosis and the deficient phagocytic clearance of the apoptotic cells together expedite plaque necrosis, which leads to atherothrombotic cardiovascular affairs (Tabas, 2010). Macrophage apoptosis and the deficient phagocytic clearance of apoptotic cells together expedite plaque necrosis, which leads to atherothrombotic cardiovascular affairs (Tabas, 2010). Oxidative stress and endoplasmic reticulum (ER) stress are mainly responsible for advanced macrophage apoptosis. Recent research has shown that pro-apoptotic oxidative stress and ER stress inducers give rise to autophagy, another stress response in the macrophages (Liao et al., 2012). The suppression of autophagy via silencing ATG5 and others autophagy mediators augments apoptosis and NOXs-mediated oxidative stress, and giving apoptotic cells fewer well-recognized by efferocytes (Li et al., 2010). Macrophage ATG5 deficiency in the Ldlr^−/−^ mice enhances oxidative stress in advanced macrophages lesion, accelerates plaque necrosis (Tabas, 2010). These results altogether uncover a mechanism in macrophages related with plaque necrosis.

### Thiol oxidative stress and oxidative stress

Thiol oxidative stress results in macrophage functional disorder, cellular damage, as well as progressed development of atherosclerotic lesions (Wang et al., 2006). Marrow cells infected with retroviral vectors were transplanted into low-density lipoprotein receptor-deficient mice (Hawley et al., 1994). After bone marrow transplantation, the animals were kept the western diet for 10 weeks. But no discrepancies in either the serum triglyceride and cholesterol levels or the macrophage contents were viewed (Qiao et al., 2007). Mouse that were reestablished with mitochondrial glutathione reducase (GRmito-EGFP) and EGFP-fusion protein of cytosolic glutathione reducase (GRcyto-EGFP)-expressing bone marrow had lesional acreage 32% fewer than those mouse who accepted EGFP-expressing, however (Qiao et al., 2007). In incubated cells, the adenovirus overexpression of GRmito and GRcyto-EGFP can preserve cellulas from the hyperpolarization of mitochondrion abduced through ox-LDL (Hawley et al., 1994). Another previous study (Qiao et al., 2007) showed that glutathione-dependent antioxidant acts a crucial effect in the atherogenesis, as well as thiol oxidative stress-abduced mitochondrial functional disorders is related with macrophage damaged in the atherosclerotic lesions.

## Inflammation induces oxidative stress in atherosclerosis

Inflammation is a crucial element in progression of atherosclerotic plaque, plaque rupture, and atherothrombosis (Cannizzo et al., 2014). The process is also important in relapsed thrombosis, where oxidative stress is given to play an important function (Freedman, 2008). Oxidative stress and inflammation are interrelated; they form a vicious feed-forward cycle during atherogenetic plaque progress (Lozhkin et al., 2017). Inflammation caused by oxidative stress seriously threatens human health (Martinon, 2010). Typical health problems include dyslipidemia (Hopps et al., 2009), metabolic syndrome (Iyer et al., 2010), and thrombosis (Leopold and Loscalzo, 2009; Xu et al., 2010). Oxidative stress activates transcription factors that alter inflammatory cytokines, soluble mediators, and chemokines. Cytokines and chemokines secreted by inflammatory cells gather inflammatory cells to the sites of inflammation, leading to increased ROS product thus exacerbating this adverse cycle (Martinon, 2010; Reuter et al., 2010). In short, oxidative stress and inflammation which are markers of atherosclerosis, promote to the progression of atherosclerosis.

### NADPH-oxidase 4 (NOX-4) and oxidative stress

Inflammation and oxidative stress are regarded as main factors accelerating angiogenesis in the early stage of atherosclerosis (Lozhkin et al., 2017). NOXs include an important and widely expressed enzyme family with ROS generation as its primary function. NOX-4 is a universally expressed in the VSMCs that are primary components of vascular wall, the functions of which are crucial determinants of vascular homeostasis and disease (Lassègue et al., 2001; Lu et al., 2013). NOX-4 mediates cardiovascular disease in hyperlipidemic mice and expression of NOX-4 in wall of the human artery is related with atherosclerotic severity (Vendrov et al., 2015). NOX-4 expression and activity during the aging process enhances cellular and mitochondrial oxidative stress, vascular inflammation, dysfunction, and atherosclerosis. Lozhkin et al. (2017) observed the enhanced expression and activation of NOX-4 in Apoe^−/−^ mice, which they ascribed to the pro-inflammatory phenotype in the VSMCs that was abduced by an age-related increase in transforming growth factor β1 thus enhancing atherosclerosis.

### Oxidized HDL (ox-HDL) and oxidative stress

HDL forfeits its cardioprotective capability due to oxidative modification through ROS in advanced atherogenesis (Xiao et al., 2015). Monocytes play a pivotal role in the atherogenesis; threfore, the effects of both native and ox-HDL in monocyte–macrophage functions related with atherogenesis are major research subjects (Soumyarani and Jayakumari, 2012; Wang et al., 2014b). Human blood monocytes were cultured under normal circumstances in the previous study (Callegari et al., 2006) to evaluate cells dealed with native HDL and ox-HDL at the diverse concentrations for different time intervals. The production of ROS was evaluated founded on the ROS-mediated dichlorodihydrofluorescein diacetate fluorescence of cells (Zhang et al., 2010). Simultaneously, the liberation of matrix metalloproteinases (MMPs) as well as tumor necrosis factors-a (TNF-a) was quantitated with an ELISA kit and gelatin zymography, respectively (Radhika et al., 2007). The HDL treatment enhanced the generation of ROS in the concentration-dependent fashion, while natural HDL cannot this action (Zhang et al., 2010; Vendrov et al., 2015). The expression of inflammatory factors was also discovered to be higher in cultured cells with ox-HDL than natural HDL (Vendrov et al., 2015). In effect, the oxidative modification of HDL abduces pro-inflammatory effects and oxidative stress in the monocyte-derived macrophagocytes during atherogenesis.

### NLRP3 inflammasome and oxidative stress

NLRP3 inflammasome takes part in the chronic inflammation under atherogenesis in the vascular walls (Duewell et al., 2010). Duewell et al. (Piedrahita et al., 1992; Duewell et al., 2010) reestablished deadly irradiated LDL receptor-deficient mice with bone marrow from mice who were then kept the high-cholesterol diet for 8 weeks. In the radiation bone marrow chimerisms, the LDL receptor-deficient radio-resistant parenchyma gave animals to be hypercholesterolemic when given the high-lipid diet. Further, these macrophagocytes and others leukocytes lacked of NLRP3-inflammasome and interleukin-1 (IL-1) pathway compositions required to cholesterol crystals of reaction (Martinon et al., 2009). The findings showed that the NLRP3 inflammasome activated through macrophages of bone marrow devotes primarily to diet-abduced atherosclerosis.

## Autophagy induces oxidative stress in atherosclerosis

Autophagy, as coined by the Belgian biochemist Christian de Duve in 1966 (De Duve and Wattiaux, 1966), is the lysosome-dependent degradation of cytoplasm and damaged cell organelles like mitochondrion, ER, and peroxisomes, along with the clearing away of intracellular pathogens (Mei et al., 2015). Autophagy is an evolutionarily preserved process through which cell organelles and intracellular proteins are sealed with double-membrane vesicles and then diverted to lysosomes and degraded (Mizushima and Komatsu, 2011). Autophagy is considered a survival mechanism (Martinet and De Meyer, 2009). Excessive autophagic activity can destruct important components, such as the organelles and cytosol, but most obviously the ER and mitochondria, ultimately resulting in the complete collapse of cellular actions and autophagic death (Levine and Yuan, 2005). Autophagy is related to CVD because it is triggered by hypoxia, inflammation, ER stress, oxidized lipoprotein, and oxidative stress which are all involved to some extent in atherogenesis (Margariti et al., 2013; Ouimet, 2013).

### Light chain 3 (LC3) and oxidative stress

In the human monocyctic THP-1 cells, autophagy-like ultrastructural characteristics through transmission electron microscopy as well as the expression of autophagy hallmarker LC3-phosphatidylethanolamine conjugate (LC3-II) through Western blot analysis (ATCC) displayed that the autophagy is a mainly component in the development of atherosclerosis (Mei et al., 2015; Yuan et al., 2016). 7-Oxysterols are major toxic components in ox-LDL and human atheromatous lesions which lead to lysosomal membrane permeabilization (LMP) and cell death (Li et al., 2001). Exposed to 7-oxysterols abduces autophagic vacuole synthesis in shape of enhanced autophagy hallmarker microtubule-related protein. In addition, autophagy induction minimizes in the cell lipid cumulation abduced by 7-oxysterols (Larsson et al., 2006). The discovery emphasize significance of autophagy in countering LMP and cell death in the atherosclerosis.

### Lectin-like ox-LDL receptor-1 (LOX-1) and oxidative stress

The ox-LDL-dependent activation of the LOX-1 causes apoptosis in cells as well as probably participates in atherosclerosis. Autophagy may effectively substitute for apoptosis in endothelial cells (Nowicki et al., 2007). Nowicki et al. (Claise et al., 1999; Nowicki et al., 2007) analyzed expression of LOX-1 and the ox-LDL-dependent action in the EA.hy926 cells amid serum starvation to discover which the serum starvation upregulates LOX-1, while other ox-LDL treatment downregulates the acceptor and enhances autophagy through increasing oxidative stress. Other researchers (Ding et al., 2013) observed intense autophagy, inflammatory signals (CD45 and CD68), as well as toll-like receptor 9 (TLR-9) expression in LDL receptor (LDLR) knockout mice raised with hyper-cholesterol diet. LDLR/LOX-1 double knockout mice decreased autophagy, CD45 and CD68, and TLR9 expression. A damaged mtDNA, which tends to be very obvious in the LDLR knockout mouse, can be reduced by LOX-1 deletion (Ding et al., 2013). To this effect, oxidative stress damaged mtDNA which escapes autophagy abduces a strong inflammatory response in the atherosclerosis.

### Autophagy-related 7 (ATG7) and oxidative stress

Autophagy is triggered in the VSMCs of diseased arterial vessels (Grootaert et al., 2015). The autophagy gene Atg7 in the Atg7^−/−^ VSMCs enhances the accumulation of SQSTM1/p62 and accelerates stress-abduced premature senescence, such as cell and nuclear hypertrophy, senescence-associated GLB1 activity, and CDKN2A-RB-mediated G1 hyperplastic block (Komatsu et al., 2005). The transfection of SQSTM1-coding plasmid DNA in the Atg7C/C VSMCs abduced semblable characteristics in another study, indicating that the cumulation of SQSTM1 promotes VSMC senility (Newby, 2006). However, compared with various controls, the Atg7^−/−^ VSMCs are accelerate to oxidative stress-abduced cell death (Komatsu et al., 2005; Newby, 2006). The function may be ascribable to the nuclear translocation of transcription factor (NFE2L2) bringing about the upregulation of some antioxidative enzymes (Sasaki et al., 2012). These studies have suggested that the defective autophagy in the VSMCs expedites the progression of oxidative stress-anduced premature senility as well as enhances the formation of atherogenesis.

## Apoptosis induces oxidative stress in atherosclerosis

Apoptosis is a form of cell death featured as cell contraction, chromatin condensation, and membrane blebbing (Kerr et al., 1972). Membrane-enclosed apoptotic cell debris is engulfed either via the surrounding cells or by phagocytes within its vicinity. Within the cardiovascular system, augmented apoptosis occurs in advanced human atherosclerotic plaques (Kockx et al., 1998; Littlewood and Bennett, 2003). Apoptosis is a crucial component in the progression of atherosclerosis. All cell genres existing in atherosclerotic plaques undergo apoptosis, embracing SMCs, lymphocytes, endotheliocytes, and macrophages (Schrijvers et al., 2005). Several known mechanisms of the oxidative stress-mediate and apoptosis in the atherosclerosis are described below.

### Granulocyte–macrophage colony stimulating factor (GM–CSF) and oxidative stress

GM–CSF is a cell growth factor involved in the pathogenesis mechanism of atherosclerosis and others inflammatory diseases (Stanley et al., 1994). A recent study (Subramanian et al., 2015) used mice raised with a western diet for 12 weeks to quantize the parameters of plaque progression in aorta. GM–CSF-deficient mice demonstrated the substantial reduce in the two crux hallmarks of the advanced atherosclerosis; this suggests that the GM–CSF boosts plaque progression (Subramanian et al., 2013). The study revealed that the mechanism involves in the GM–CSF-mediated generation of the IL-23, where adds apoptosis sensitivity to macrophages through increasing the proteasomal degradation of the cell-survival protein B-cell lymphoma-2 (Bcl-2) along with oxidative stress in the LDL-driven atherosclerosis (Tausend et al., 2014).

### Protein kinase Cβ (PKCβ) and oxidative stress

Protein kinase Cβ (PKCβ), a membership of PKC family of the serine-threonine protein kinases, is given to be a crucial pro-apoptotic signal in numerous cell genres (Larroque-Cardoso et al., 2013). In atherogenesis, exorbitant LDL accumulate in subendothelial space which they play sorts of oxidized modifications (Reyland, 2007). The ox-LDL influence the vulnerable balance between survival and death in cells, resulting in plaque instability leading to atherothrombotic events (Salvayre et al., 2002). PKCβ is pro-apoptotic in numerous cell genres; a recent study (Larroque-Cardoso et al., 2013) was conducted to survey its latent action in regulation of VSMC apoptosis abduced by ox-LDL. Human VSMC silenced for PKCβ was effectually protected against ox-LDL-induced apoptosis, and PKCβ activation hinged on the ROS produced by ox-LDL (Salvayre et al., 2002; Reyland, 2007). The same study also indicated that PKCβ takes part in the ox-LDL-abduced apoptotic signaling primarily via IRE1a/JNK pathway.

### Haptoglobin 2-2 (Hp2-2) plaques and oxidative stress

Intraplaque hemorrhage liberates free hemoglobin (Hb) (Levy et al., 2007). Damaged Hb clearance causes the oxidative stress resulting in the plaque formation (Asleh et al., 2005). Combining of Hp to Hb decreases iron-abduced oxidative responses (Asleh et al., 2005; Levy et al., 2007). A total of 26 populations aortic plaques were Hp-genotyped in the previous study to compare Hp2-2 plaques with the control plaques (Hp1-1/2-1) according to their respective iron levels measured through Perl's staining (Purushothaman et al., 2012); and immunostaining was employed to test oxidation-specific epitopes (OSEs) mirroring malondialdehyde (MDA) epitopes and oxidized phospholipids. In the study, the active caspase-3 and DNA fragmentation were surveyed, respectively (Purushothaman et al., 2012). These outcomes have provided notable insight into the genetic lean to oxidative stress and the correlation both macrophage apoptosis and OSEs related with advanced atherosclerosis in the human Hp2-2 plaques.

### B-cell lymphoma-2 (Bcl-2) and oxidative stress

The Bcl-2 gene maybe significant in regards to the formation of atherosclerotic plaques (Zurgil et al., 2007). Apoptosis in pathophysiology of atherosclerosis had indicated by powerful relevance between Bcl-2 protein and apoptosis in the progression of atherosclerotic, as well as the suppression of ox-LDL-abduced apoptosis through the Bcl-2 protein (Wang et al., 2001) and Bax expression within human fatty streaks (Hata et al., 2001). A clinical study (Zurgil et al., 2007) on lymphocytes acutely isolated from 25 angina sufferers and 27 healthy donors were tested to assess in apoptotic affairs educed by lysophosphatidylcholine (LPC) in the static and phytohemagglutinin (PHA)-activated lymphocytes, as well as to gauge the expression of the Bax and Bcl-2 and levels of intracellular ROS (Wang et al., 2001). LPC was found to abduce apoptosis with augmenting levels of the intracellular ROS. The exposure of the PHA-activated PBL to LPC was correlated to a markedly lower expression of Bax/Bcl-2 ratio (Wang et al., 2001; Zurgil et al., 2007). Oxidative stress concerned to apoptosis-associated protein expression led to undue or altered cell and immune responses in diverse stages of atherogenesis.

### Superoxide dismutase (SOD) and oxidative stress

The increased sensitivity of monocytes to ox-LDL-induced oxidative stress may be attributed to the concomitant overexpression of SOD in monocytes undergoing apoptosis. Zurgil et al. (2004) used the mechanism of cell death in 2-model systems, T lymphocytes and monocytic cell line exposed to ox-LDL. Apoptotic cell death was analyzed by evaluating cell size, nucleic DNA content, and plasma membrane asymmetry. The radical scavenger SOD declined the apoptotic effects of the ox-LDL in the time-dependent and dose-dependent styles (Kinscherf et al., 1998; Zurgil et al., 2004). Ox-LDL binding also activates the macrophages and monocytes and irritates SOD expression, which enhances concentrations of hydrogen peroxide through interfered ROS levels (Kinscherf et al., 1997, 1998). The process is correlated to a great deal of macrophage apoptosis bringing about atherosclerotic lesion (Reid et al., 1993).

## Mitochondria induce oxidative stress in atherosclerosis

Mitochondria are dynamic organelles in eukaryotic cells with heterogeneous morphology that is dominated by the equilibrium created by alternating fission and fusion (Chang et al., 2010). The dynamic nature of mitochondria includes the domination of its architecture (distribution and morphology), its movement across the cytoskeleton, and the connectivity mediated by restraining and fusion/fission events (Liesa et al., 2009). Mitochondrion and nonphagocytic NOXs are main sources of chronic ROS generation beneath physiological conditions (Luft and Landau, 1995; Sorescu and Griendling, 2002). And enhanced mitochondrial ROS generation and functional disorder are correlated to CVD and numerous other diseases (Gropen et al., 1994; Anan et al., 1995; Wallace, 1999). Furthermore, mitochondrial dysfunction is conducive to the development of atherosclerosis as-evidenced by animal and human models of oxidative stress (Madamanchi and Runge, 2007, 2013). Mitochondrial dysfunction can also boost these pro-atherogenic processes; mitochondrial damage participates in atherogenesis by mtDNA damage (Ballinger et al., 2002). Some substances actively participate in the mitochondrial oxidative damage and accelerate atherosclerosis progression described as follows.

### Retinol-binding protein 4 (RBP4) and oxidative stress

RBP4 is the sole carrier of retinols, and is hence takes charge of the transport of retinol from liver storages to peripheral tissues (Blaner, 1989; Newcomer and Ong, 2000). Serum RBP4 increasing has been connected to cardiovascular system disease. Researchers (Wang et al., 2015) found a mechanism by which RBP4 causes the oxidative stress of blood vessels and promotes the pathogenesis of atherosclerosis. In another study, RBP4 therapy enhanced superoxide production in the dose-dependent way in the human aortic endothelial cells (HAECs) (Blaner, 1989; Newcomer and Ong, 2000; Wang et al., 2015). Exposed to RBP4 also expedited mitochondrial function disorder, as-ascertained by lessened mitochondrial contents and completeness as well as membrane potential (Wang et al., 2014a). The RBP4 stimulation restrained protein kinase B signaling in the HAECs (Wang et al., 2015). The RBP4-Tg mice also showed serious vascular oxidative injure as well as mitochondrial function disorder in aorta, compared with widespread-type C57BL/6J mice (Blaner, 1989; Newcomer and Ong, 2000; Wang et al., 2015).

### Macrophage mitochondrial oxidative stress (mitoOS)

A previous study investigated the significance of macrophage-mitoOS using mitochondrial catalase (mCAT) transgenic mice and Ldlr^−/−^ mice in which the oxidative stress suppressor catalase was expressed in mCAT in macrophages (Wang et al., 2014a). MitoOS in lesional macrophages was markedly suppressed in these mice, bringing about a notable reduction in aortic damaged zone (Moore and Tabas, 2011). mCAT lesions had fewer monocytes, lower levels of the monocyte chemotactic protein-1 (MCP-1), as well as less Ly6chi monocyte infiltration into the lesions (Wang et al., 2014a). Reduction in damaged MCP-1 was attributed to the inhibition of others inflammation markers as well as reduced nuclear factor-k-gene binding (NF-κB), suggesting reduced activity of the inflammatory NF-κB pathway (Moore and Tabas, 2011; Wang et al., 2014a). In incubated macrophages employing models of mitoOS, the results found that the mCAT inhibited the expression of MCP-1 through reducing the activity of Iκ-kinase-RelA NF-κB pathway (Schriner et al., 2005). These results suggest that the mitoOS in injury macrophages enhances the progression of atherosclerotic through accelerating the NF-κB-mediated access of monocytes and others processes of inflammatory (Schriner et al., 2005; Moore and Tabas, 2011; Wang et al., 2014a).

### Mitochondrial genome (mtDNA) and oxidative stress

In the pathology, some illnesses are related to mutations in mtDNA (Sobenin et al., 2013). Mitochondrial function disorder creates favorable qualifications for the pathogenesis of atherosclerosis (Madamanchi and Runge, 2007, 2013). Researchers (Sazonova et al., 2009; Sobenin et al., 2013) have studied that relation of mitochondrial gene mutation with the severity of atherosclerosis in 190 sufferers from Russia in particularly high coronary heart disease (CHD) prevalence. The cIMT was detedted by mtDNA heteroplasmy and B-mode ultrasonography through pyrosequencing technique (Sazonova et al., 2009). The results manifested that mitochondrial gene mutation play a part in the development of atherosclerosis.

### Homocysteine (Hcy) and oxidative stress

Hcy was found to induce endothelial function disorder and atherosclerosis through ROS production (Austin et al., 1998). Meanwhile, Hcy-abduced ROS in the endotheliocytes can result in enhanced mitochondrial dysfunction (Kanani et al., 1999). The previous study (Perez-de-Arce et al., 2005) showed that Hcy-abduced ROS results in the NF-κB activity as well as enhances the formation of 3-nitrotyrosine (3-NT). In addition, the levels of nuclear respiratory factor-1(NRF-1), mitochondrial biogenesis factor, as well as mitochondrial transcription factors A (Tfam) expression were notably enhanced in the Hcy-handled cells (Perez-de-Arce et al., 2005). And these variations were accompanied with an augmentation in the mitochondrial quality and the mRNA contents, and enhance in levels of protein expression of cytochrome c oxidase subunit III (Kanani et al., 1999; Perez-de-Arce et al., 2005). These influences were effectively protected from pretreatment with antioxidants as well as catechin. In short, the ROS is a significant mediator of the mitochondrial dyfunction ebduced by Hcy.

## Angiotensin II (Ang II) and oxidative stress

Ang II induced oxidative stress participates in the development of atherosclerosis (Mehta and Griendling, 2007). At the same time, this signaling pathway CD40/CD40L plays a significant part in the progression of atherosclerotic plaque formation and rupture (Law et al., 1990). Previous researchers (Souza et al., 2009) tested the hypothesis that Ang II enhances the CD40/CD40L activated in the angiocellulars, as well as that ROS is a portion of the signaling cascade which regulates expression of CD40/CD40L. In incubated human coronary artery smooth muscle cells, exposed of TNF-α or IL-1 beta exhibit enhanced superoxide production and increased expression of CD40 which can be confirmed by immunoblotting and electron paramagnetic resonance analyses (Law et al., 1990; Souza et al., 2009). The Ang II stimulus of angiocellulars results in an ROS-dependent enhancement in the activity of CD40/CD40L signaling pathway during atherosclerosis.

## Epigenetic, DNA methylation, and histone modification induce oxidative stress in atherosclerosis

Epigenetics refers to sorts of dynamic characteristics that modify genomic function under exogenous impact and offer a molecular substrate that permits the steady reproduction of the gene expression statuses from one generation cells to the next (Feinberg, 2008). Epigenetic modifications, such as histone modifications and DNA methylation, appear to play an importantly effect in the processes underlying atherosclerosis (Borghini et al., 2013). Atherosclerosis begins a focal disease resulting from complicated gene-surroundings interplays (Lusis, 2012). Epigenetics describes phenomena connected to the expression of heritable information independent of changes in DNA sequence. DNA methylation reflects altered functions of cell types participating in immune or inflammatory reactions during atherosclerosis (Zaina et al., 2005). Following previsional early validations of the flow actions upon chromatin remodeling (Illi et al., 2003) and histone encoding (Fish et al., 2005), others several mechanisms like the DNA methyltransferases (Dunn et al., 2014; Jiang et al., 2014; Zhou et al., 2014) and the microRNAs (Fang et al., 2010; Zhou et al., 2011; Fang and Davies, 2012; Kumar et al., 2014) are known to adjust the flow-sensitive endothelial phenotype.

### Ox-LDL and oxidative stress

Ox-LDL exposure induces various functions in the endotheliocytes, such as the liberation of cytokines, chemotactic factors, and growth factors, as well as the expression of the surface molecules which adjust hemostatic properties and endothelial permeability; it is also associated with variations in the cell proliferaton, division, and apoptosis (Navab et al., 2004; Lahoute et al., 2011). A recent study (Yang et al., 2014) suggested that in HCAECs treated with ox-LDL alone, cell viability, DNA synthesis, as well as the expression of promoted survival fibrocyte growth factor 2 (FGF2) markedly decrease. The suppression impacts of ox-LDL were observably decreased in the HCAECs co-treated with the anti-malondialdehyde (anti-MDA) (Yang et al., 2014). The study assessed the influences of a group of the regulators on the signal transduction pathways of the MDA in ox-LDL-dealed HCAECs to discover that MDA-abduced cell toxicity is mediated partly via the Akt pathway (Yang et al., 2014). These outcomes of genome DNA sequencing suggested that treated with ox-LDL in the HCAECs, GC promoter of FGF2 was methylated at the cytosine residues and that co-treatment with anti-MDA significantly decreased ox-LDL-abduced FGF2 promoter methylation (Valko et al., 2006; Yang et al., 2014). These results suggest that ox-LDL destroys the growth process of the HCAECs via the MDA-dependent pathway about the suppression of the FGF2 transcription as well as the methylation of FGF2 promoter (Navab et al., 2004; Valko et al., 2006; Lahoute et al., 2011; Yang et al., 2014). The recently realized pathogenesis of epigenetics may underlie atherosclerosis in the subjects with cardiovascular system disease.

### DNA methylation and oxidative stress

**Figure 2:**
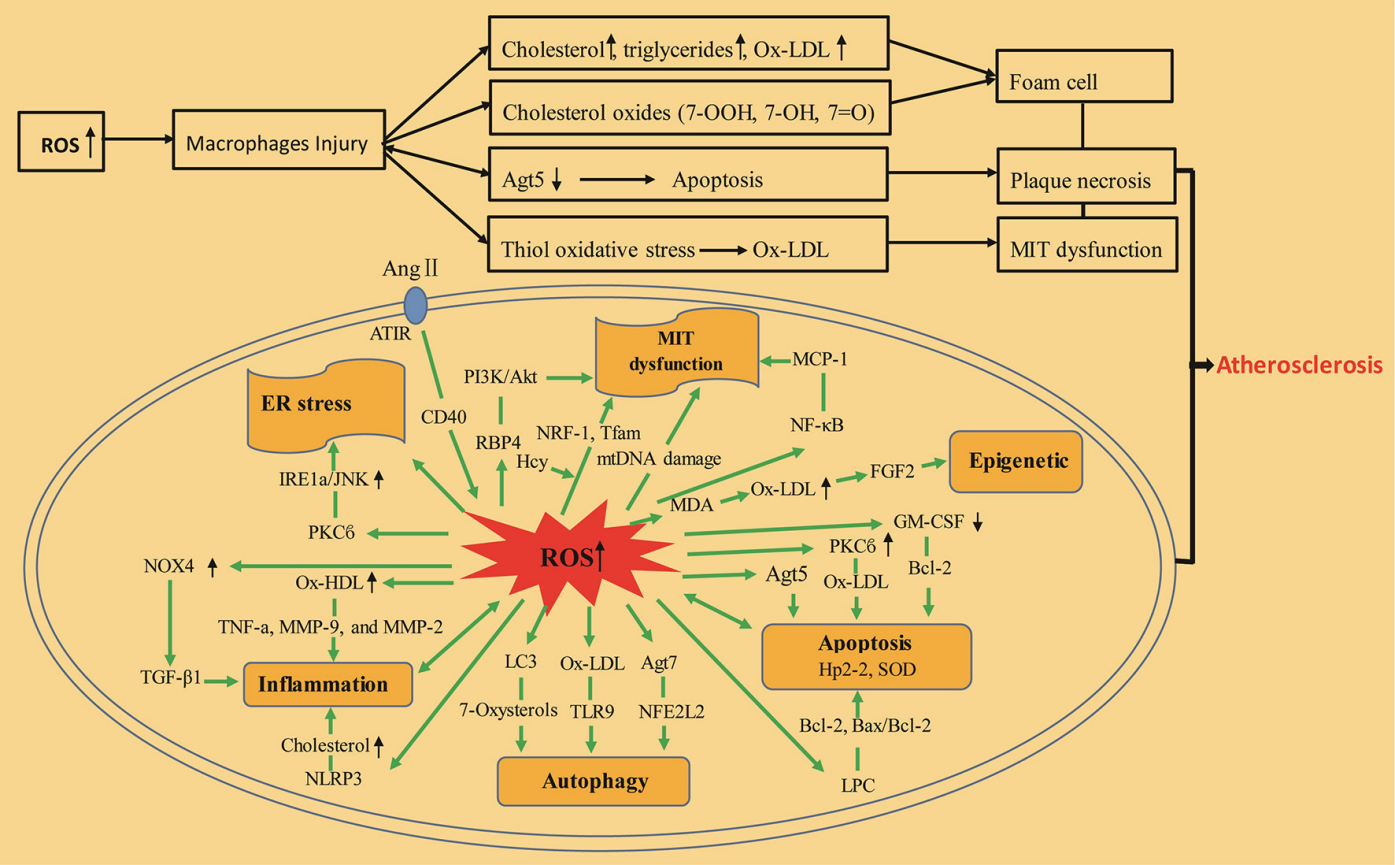
Inflammation, mitochondria, autophagy, apoptosis, and epigenetics-induced oxidative stress during atherosclerosis. ox-LDL, oxidized low-density lipoprotein; ROS, reactive oxygen species; 7-OOH, 7-hydroperoxide; 7-OH, 7-hydroxide; 7 = O, 7-ketone; ATG5, autophagy protein 5; PKCß, protein kinase Cß; ox-HDL, oxidized high-density lipoprotein; ER stress, endoplasmic reticulum stress; TGF-β1, transforming growth factor β1; NOX-4, nicotinamide adenine dinucleotide phosphate (NADPH)-oxidase 4; TNF-a, tumor necrosis factors-a; MMP-9, matrix metalloproteinase-9; NLRP3, Nod-like receptor pyrin domain-containing protein 3; MMP-2, matrix metalloproteinase-2; LC3, light chain 3; TLR-9, Toll-like receptor 9; NFE2L2, nuclear translocation of the transcription factor; ATG7, autophagy-related 7; GM–CSF, granulocyte–macrophage colony stimulating factor; Hp2-2, haptoglobin 2-2; Bcl-2 and Bax, apoptotic regulatory proteins; LPC, lysophosphatidylcholine; SOD, superoxide dismutase; Ang II, angiotensin II; ATlR, angiotensin-converting enzyme receptor 1; RBP4, retinol-binding protein 4; MCP-1, monocyte chemotactic protein-1; Hcy, homocysteine; NF-kB, nuclear factor-k-gene binding; Txnip, thioredoxin-interacting protein; NRF-1, nuclear respiratory factor-1; PI3K/AKT, phosphatidylinositol 3 kinase/protein kinase B; Tfam, mitochondrial transcription factor A; MIT dysfunction, mitochondrial dysfunction; mtDNA damage, mitochondrial DNA damage; FGF2, fibroblast growth factor 2; MDA, malondialdehyde.

Atherosclerosis is an artery disease of heterogeneous distribution in which endothelium acts a significant central effect (Lusis, 2012). A recent study (Jiang et al., 2015) showed methylome blueprint for the spatio-temporal analysis of the lesion susceptivity induced to endothelial functional disorder in the complicated flow circumstances in the vivo. Exposed to particulate air contamination had also been associated with enhanced death, especially in cardiovascular system disease (Jiang et al., 2015). And lower DNA methylation contents had been observed in the process linked to cardiovascular events, as oxidative stress and atherosclerosis (Baccarelli et al., 2009; Jiang et al., 2015). Baccarelli et al. (2009) researched DNA methylation in scattered nucleotide element-1 and Alu repeating elements via the pyrosequencing of 1,097 specimens from 718 patients in Boston. Other researchers employed covariate-regulated mingled models to explain the within-patient relevance in the duplicated measures; reduced duplicated-element methylation was observed after exposed to traffic-related pollutant granules (Baccarelli et al., 2009). This finding elucidated the role of DNA damage and mending in the pathogenesis of atherosclerosis and the connection to epigenetic modifications (Figure 2).

## Therapeutic effect of antioxidants in atherosclerosis

Antioxidant defense systems are the main material basis for protection against free radicals. They can eliminate free radicals before they attack target cells by preventing cellular damage and monitoring the concentration of free radicals throughout the body. As discussed above, oxidative stress is related with the formation and development of the atherosclerotic plaques. As a consequence, antioxidant therapy is an ordinary ways to atherosclerosis treatment (Table 1). Clinical trials on antioxidant therapy have been generally unsuccessful, though probucol, the most powerful antioxidant, has been found to inhibit ox-LDL, delay atherosclerosis progression, and reduce the occurrence of vascular events. Angiotensin-converting enzyme inhibitors (ACEI), vitamins, angiotensin receptor antagonists, calcium antagonists, as well as statins can effectively supress NOX activity and mitigate oxidative stress (Paravicini and Touyz, 2008).

**Table 1:** Main antioxidants and mechanisms.

**Antioxidant**	**Mechanism**	**References**
Vitamin	Protecting against oxidative damage induced by hydrogen peroxide.	Luft and Landau, 1995; Kinscherf et al., 1997; Liesa et al., 2009; Chang et al., 2010
ACEI	Increasing plasma bradykinin to diastolic coronary vessels and peripheral blood vessels.	Gropen et al., 1994
ATlR antagonists	Blocking the action of Ang II; blocking ROS production from the source; inhibiting the expression of vascular endothelial cells.	Anan et al., 1995
Statins	Increasing NO bioactivity; upregulating NOS expression.	Blaner, 1989; Ballinger et al., 2002; Madamanchi and Runge, 2007
Probucol	Reducing plasma oxygen free radical concentration; inhibiting LDL formation.	Wang et al., 2014a, 2015
AGI-1067	Protecting the vascular system with antioxidant properties.	Schriner et al., 2005; Sobenin et al., 2013

*ACEI, angiotensin-converting enzyme inhibitors; ATlR antagonists, angiotensin-converting enzyme receptor 1 antagonists; AGI-1,067, succinobucol; NO, nitric oxide; NOS, nitric oxide synthase; LDL, low-density lipoprotein*.

### Antioxidant vitamins

Previous studies have obviously elucidated the function of oxidative stress in the development of diseases. Numerous proofs manifests that increased lipid oxidation expedites atherogenesis (Salonen et al., 1992, 1997; Gaut and Heinecke, 2001; Witztum and Steinberg, 2001), as well as that the employ of antioxidant supplements decreases atherosclerosis (Azen et al., 1996; Gale et al., 2001). The study suggested that vitamin E and vitamin C are the most significant dietary antioxidation treatment (Frei et al., 1989; Diaz et al., 1997); when vitamin E serves as an antioxidant, it is oxidized to pernicious radicals and have to be decreased back to tocopherol through secondary supplements, such as vitamin C (Packer et al., 1979). Wang (2005) found that vitamins C and vitamins E protected oxidative injure abduced by hydrogen peroxide in the vascular endothelial cells. These two vitamins maintain cell morphology, reduce lipid peroxidation, and improve anti-lipid peroxidation.

Recent large-scale clinical tests (Salonen et al., 2003) have yielded hopeful results (Table 2). Bleys et al. (2006) conducted a meta-analysis indicating that vitamin supplement therapy cannot prevent the atherosclerosis progression, however. Further, both Lee et al. (2005) and Sesso et al. (2008) failed to prove that vitamin consumption benefits cardiovascular endpoints via randomized controlled trials including middle-aged women and men. That being said, several small clinics have reported that orally administered vitamins improve vascular endothelial function by reducing the occurrence and progress of atherosclerosis (He, 2009). Disparities in results across these studies may be attributed to antioxidant limitations. The extant antioxidant atherosclerosis research is overwhelmingly negative; the use of drugs is usually inconclusive and relevant guidelines do not include vitamin antioxidants as treatment for CVD.

**Table 2:** Main clinical trials on antioxidant vitamin therapy.

**Study**	**Participants (patients)**	**Intervention**	**Follow-up (year)**	**Outcomes**
CHAOS	2,002	Vitamin E (800 mg/d) or (400 mg/d)	15	Reduces cardiovascular events, but cardiovascular mortality is not obvious.
HPS	20,536	Vitamin E (600 mg/d), Vitamin C (250 mg/d), and β-carotene (20 mg/d)	5	Slightly affects mortality and risk.
ASAP	946	Vitamin E (136 mg) plus Vitamin C (250 mg) twice daily	3	Slows down atherosclerotic progression in hypercholesterolemic individuals.
PPP	4,784	Vitamin E (300 mg/day)	5	Vitamin E effect is not obvious.

*CHAOS, Cambridge Heart Antioxidant Study; HPS, Heart Protection Study; ASAP, Antioxidant Supplementation in Atherosclerosis Prevention; PPP, Primary Prevention Project*.

### ACEI and ATLR antagonists

ACEI is an angiotensin converting enzyme inhibition that guards against the generation of Ang II, thus preventing vessel contraction, stimulating aldosterone liberate, and enhancing blood volume, blood pressure, and nitric oxide generation. It can also protect vascular endothelial cells and improve cardiomyocyte resistance to free radical damage. Finally, it can effectively prevent atherosclerosis, reduce the incidence of cardiovascular events, and treat atherosclerosis with a wide range of applications.

ACEI and ATlR antagonists are commonly used to treat coronary atherosclerotic heart disease. Ang II has been demonstrated to activate NOXs and XO, enhancing ${\text{O}}_{2}^{•-}$ production and promoting ROS (Raffaele and Peter, 2007). ACEI and ATlR antagonists may block the effect of Ang II, effectually blocking the origin of ROS generation, thereby inhibiting endothelial cells as well as decreasing atherosclerotic factor expression.

### Statins

Statins can decrease the risk of relapsed cardiovascular affairs by 30% (Akdim et al., 2007) as well as are the major source of medications for atherosclerosis. They not only reduce the lipid-lowering role of medications, but also participate in the adjustment of cellular proliferation, intracellular signal transduction, and others functions; they prevent inflammatory response, improve endothelial function, block the formation of foam cells, and inhibit anti-platelet aggregation (Chapman, 2007). Statins also inhibit certain coagulation factors and MMPs in the product of unstable plaques (Chapman, 2007). In fact, high-strength statin treatment can effectively prenent plaque progression.

Hypercholesterolemia can activate ROS, reducing nitric oxide activity and inducing endothelial dysfunction. Statins can reduce cholesterol and act on vascular cell NADPH oxidase to increase the endothelial function. Statins also regulate lipid metabolism and can inhibit NADPH oxidase-induced ${\text{O}}_{2}^{•-}$. Hao et al. (2005) found that simvastatin could obviously enhance the activation of antioxidative enzymes submited as increases in the SOD and GPx and a reducing in MDA levels in a hyperlipidemia rabbit model, thereby improving the antioxidant capability of the body and postponing the progression of atherosclerosis. The Familial Atherosclerosis Treatment Study demonstrated the significant efficacy of lovastatin combined with colestipol for atherosclerosis (Brown et al., 1990). In addition, the findings of the University of California Special Centre of Research Trial and Monitored Atherosclerosis Regression Study strongly supported the beneficial effects of statin therapy (Blankenhorn et al., 1993).

### Probucol and AGI-1067 (succinobucol)

Probucol was first listed as a lipid-lowering drug in the United States in 1977. It is currently recognized as the most promising and effective first-line antioxidant in the treatment of atherosclerosis. The drug exerts a strong antioxidant effect originating mainly from oxygen ion capture and chain-breaking properties (Tardif, 2003). Probucol molecules containing phenolic hydroxyls are readily oxidized and break open, capturing oxygen ions and combining to form a stable phenoxy group which reduces plasma oxygen free radical concentration and inhibits LDL formation. Russell et al. (1998) observed that blood lipid levels in patients who received probucol were not significantly reduced relative to those in a control group; however, arterial and myocardial ischemic damage was markedly reduced compared to the control, showing suggesting that the protective function of probucol is indeed its antioxidant effects. Said effects primarily happen at the blood vessel endothelium level and are showed as significant suppression of macrophages in the endothelial surface adhesion, which boosts endotheliocyte as well as smooth muscle cell functionality. Yan et al. (2003) also discovered that probucol markedly restrains protein secretion and gelatin degradation activity in the MMP-9 abduced by ox-LDL in the THP-1 cellulas in the absence of the cell activity. Probucol may increase the secretion and activity of monocyte-macrophage MMP-9 in the plaque, reducing collagen degradation in the plaque and stabilizing it to prevent atherosclerosis.

AGI-1067 is a stable analog for probucol as a vascular protective agent with equivalent antioxidant properties. Kunsch et al. (2004) found that anti-inflammatory and anti-atherosclerotic roles of the AGI-1067 are connected with their selective suppression of redox-sensitive gene expression in endotheliocytes and monocytes; probucol also inhibits *in vitro* human endothelial cell basal active oxygen cluster levels, the level of young monocyte lines, and hydrogen peroxide-induced ROS. Sundell et al. (2003) conducted animal experiments to find that AGI-1067 in kiwi, L-deficient mice, and ApoE-deficient mice suppresses the progression of atherosclerosis because of lipid-lowering functions and anti-inflammatory functions. Phase III trial of aggressive reduction of inflammation stops events manifested that the AGI-1067 can decrease the morbidity of stroke and myocardial infarction in sufferers in atherosclerosis (Tardif et al., 2008).

## Conclusion

Cardiovascular and cerebrovascular diseases caused by atherosclerosis present a serious threat to human health worldwide. Oxidative stress is the focus of most studies on atherosclerosis. The extant literature clearly demonstrates that elevated ROS levels resulting in angiooxidative stress act a crucial mechanical effect in the devolepment of atherothrombotic (Leopold and Loscalzo, 2009). For this reason, clearing the body ROS or increasing antioxidant capacity are deemed key points in the precaution and therapy of atherosclerosis; however, the precise mechanisms have yet to be elucidated. In this review, we discussed the effects of macrophages, inflammation, mitochondria, autophagy, apoptosis, and signaling pathways on oxidative stress in atherosclerosis. Recent studies have revealed a number of potentially effective treatments for atherosclerosis-related diseases. Contrary to some clinical results on antioxidant treatments, probucol and AGI-1067 have shown notable potential for application. The atherosclerosis research community has developed a more comprehensive understanding of oxidative stress, and antioxidant therapy is likely to emerge as an effective approach to atherosclerosis treatment.

## Author contributions

HS and YX defined the research theme. YL, YdL, and XR searched for related articles. XZ, DH, YG, and XY collated all related articles. XY wrote the manuscript. All authors commented on the manuscript.

### Conflict of interest statement

The authors declare that the research was conducted in the absence of any commercial or financial relationships that could be construed as a potential conflict of interest.
